# Quality of Breast Cancer Treatment at a Rural Cancer Center in Rwanda

**DOI:** 10.1200/JGO.2016.008672

**Published:** 2017-05-12

**Authors:** Daniel S. O’Neil, Nancy L. Keating, Jean Marie V. Dusengimana, Vedaste Hategekimana, Aline Umwizera, Tharcisse Mpunga, Lawrence N. Shulman, Lydia E. Pace

**Affiliations:** **Daniel S. O’Neil**, Columbia University Medical Center, New York, NY; **Nancy L. Keating** and **Lydia E. Pace**, Brigham and Women’s Hospital and Harvard Medical School, Boston, MA; **Jean Marie V. Dusengimana**, Partners in Health/Inshuti Mu Buzima, Rwinkwavu; **Vedaste Hategekimana**, **Aline Umwizera**, and **Tharcisse Mpunga**, Ministry of Health, Kigali, Rwanda; **Lawrence N. Shulman**, Abramson Cancer Center, University of Pennsylvania, Philadelphia, PA.

## Abstract

**Purpose:**

As breast cancer incidence and mortality rise in sub-Saharan Africa, it is critical to identify strategies for delivery of high-quality breast cancer care in settings with limited resources and few oncology specialists. We investigated the quality of treatments received by a cohort of patients with breast cancer at Butaro Cancer Center of Excellence (BCCOE), Rwanda’s first public cancer center.

**Patients and Methods:**

We reviewed medical records of all female patients diagnosed with invasive breast cancer at BCCOE between July 2012 and December 2013. We evaluated the provision of chemotherapy, endocrine therapy, surgery, and chemotherapy dose densities. We also applied modified international quality metrics and estimated overall survival using interval-censored analysis.

**Results:**

Among 150 patients, 28 presented with early-stage, 64 with locally advanced, and 53 with metastatic disease. Among potentially curable patients (ie, those with early-stage or locally advanced disease), 74% received at least four cycles of chemotherapy and 63% received surgery. Among hormone receptor–positive patients, 83% received endocrine therapy within 1 year of diagnosis. Fifty-seven percent of potentially curable patients completed surgery and chemotherapy and initiated endocrine therapy if indicated within 1 year of biopsy. Radiotherapy was not available. At the end of follow-up, 62% of potentially curable patients were alive, 24% were dead, and 14% were lost to follow-up.

**Conclusion:**

Appropriate delivery of chemotherapy and endocrine therapy for breast cancer is possible in rural sub-Saharan African even without oncologists based on site. Performing timely surgery and ensuring treatment completion were key challenges after the opening of BCCOE. Further investigation should examine persistent quality gaps and the relationship between treatment quality and survival.

## INTRODUCTION

Breast cancer incidence and mortality are increasing in low- and middle-income countries (LMICs). In sub-Saharan Africa, incidence has risen by approximately 30% in the past two decades.^1^ With advanced presentations and limited access to high-quality treatment, breast cancer outcomes in LMICs, including sub-Saharan Africa, are far inferior to outcomes in the United States.^2^

There are few studies examining the quality of breast cancer care in sub-Saharan Africa, but significant barriers to effective care clearly exist. Women typically present with advanced cancers.^3-5^ Pathology services are sparse and sometimes of low quality.^6^ Access to mastectomy can be limited; chemotherapy is underused and often incomplete.^7-11^ More than half of African countries have no radiotherapy capacity.^12^ Patients often travel far from home for care or cannot afford indicated treatment.^13-15^

Successful strategies for management of breast cancer in sub-Saharan Africa are urgently needed. The Butaro Cancer Center of Excellence (BCCOE) in Rwanda is one facility using an innovative model to address some of these challenges. BCCOE was established by the Rwandan Ministry of Health in collaboration with the international nongovernmental organization Partners in Health and with Dana-Farber Cancer Institute (Boston, MA). BCCOE opened in July 2012 within a rural district hospital and serves as the primary center for the Rwandan public cancer care system. Most care is provided by local and international generalists, internists, and pediatricians using evidence-based and contextually relevant protocols. Remotely based oncologists provide support via teleconferences and e-mail. Specialized nursing and pharmacy services are provided by the local Ministry of Health staff (with additional training) as well as by international volunteers. Rwanda has a national health insurance program with sliding-scale fees, and cancer treatment at Butaro is additionally subsidized through philanthropic support. Patients do not pay for chemotherapy or other cancer-specific care. Additional financial support (eg, for transport costs) is available to patients who are especially vulnerable.^16^

Rigorous assessments of the quality of breast cancer care and outcomes at BCCOE are needed to guide program improvement and understand its potential as a model for cancer care in similar settings. However, most available breast cancer quality measures have been developed for high-resource countries.^17-20^ Context-appropriate measures could guide assessment at BCCOE and be adapted to similar settings.^21^

In this study, we investigated the quality of breast cancer care provided during the first 3 years after the opening of BCCOE using a set of metrics relevant to BCCOE, including evidence-based breast cancer care quality measures adapted from measures used in high-income countries, measurements of chemotherapy dose-intensity, assessment of treatment completion, and early overall survival rates. We also hoped to refine our understanding of the utility of such measures in assessing the quality of breast cancer care in Rwanda and similar settings.

## PATIENTS AND METHODS

### Patients and Data Sources

Our retrospective cohort included all women with pathologically confirmed invasive breast cancer diagnosed at BCCOE between July 1, 2012, and December 31, 2013. Data on demographic and clinical characteristics, treatment, and outcomes were collected from patient records.

### Available Diagnostic and Treatment Services

**Fig A1:**
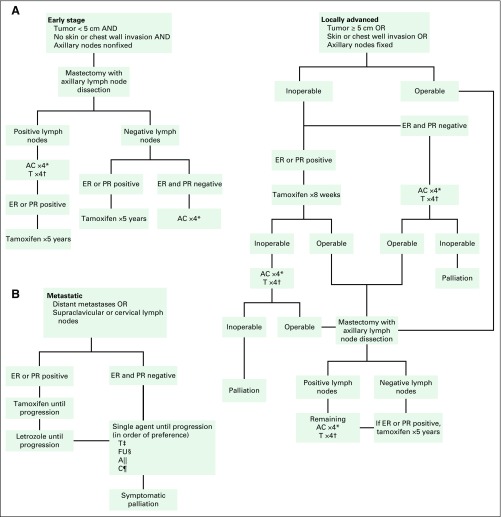
Treatment outline for (A) disease localized to breast and axilla and (B) metastatic disease beyond axillary lymph nodes or disease that is surgically unresectable despite systemic therapy. A, doxorubicin; C, cyclophosphamide; ER, estrogen receptor; FU, fluorouracil; PR, progesterone receptor; T, paclitaxel. (*) A 60 mg/m^2^ and C 600 mg/m^2^ every 21 days. (†) T 175 mg/m^2^ every 21 days. (‡) T 80 mg/m^2^ every 7 days or 175 mg/m^2^ every 21 days. (§) FU 500 mg/m^2^ every 7 days with leucovorin. (‖) A 20 mg/m^2^ every 7 days or 60 mg/m^2^ every 21 days. (¶) C 600 mg/m^2^ every 21 days.

Breast cancer services at BCCOE include biopsy, pathology evaluation, chemotherapy, endocrine therapies, and basic diagnostic radiology. During our study period, breast biopsies were taken via core needle or incision. Samples were fixed in formalin and by 2013 had been processed into paraffin blocks on site using a standard protocol to minimize tissue ischemia time. Pathologists at Brigham and Women’s Hospital (Boston, MA) analyzed the blocks, including performing immunohistochemistry analysis. Now immunohistochemistry is available at BCCOE, and pathologic interpretations are made by an onsite pathologist.^22^ Breast surgery has been intermittently available on site; otherwise, patients are referred to other national hospitals. Radiotherapy and human epidermal growth factor receptor 2 (HER2) –targeted therapies are not available. Curative-intent chemotherapy consists of doxorubicin and cyclophosphamide combination therapy followed by paclitaxel. Single-agent chemotherapy is used for palliation (Appendix Fig A1, online only).

### Key Variables

Patients at BCCOE were staged based on physical examination, chest x-ray, and abdominal ultrasound. BCCOE protocols grouped patients as having early-stage, locally advanced, or metastatic disease. For this analysis, we defined patients meeting criteria for American Joint Committee on Cancer, seventh edition, stage I to II disease as having early-stage disease, stage IIIa to IIIb as having locally advanced disease, and stage IIIc to IV as having metastatic disease.^23^ Patients with early-stage or locally advanced disease were regarded as potentially curable.

For chemotherapy, we collected administration dates, doses, and reasons for delay or dose reduction. We captured chemotherapy adverse events, but documentation was insufficient to grade severity except in the case of neutropenia. We also recorded endocrine therapies prescribed and dates initiated. Endocrine therapy adverse events were sparsely documented and are not reported.

Data on surgery date and type were collected but were sometimes unavailable. If only the month of surgery was known, the midpoint of the month was used as the estimated date of surgery. If no date was available, the midpoint between the last presurgery and first postsurgery visits was used. A surgical pathology report describing lymph nodes or provider documentation of modified radical mastectomy or axillary lymph node dissection (ALND) was considered evidence that ALND was performed.

We recorded the date of death for decedents and the last known date alive for all patients. Date of death was recorded in the medical record if a patient died at BCCOE. However, when death was confirmed by a family member via telephone, sometimes only the month and year of death were recorded in the medical record. If no timing information was documented, death was only known to have occurred between the last visit and the day of telephone contact.

### Quality Measures

We adapted breast cancer quality measures previously developed for the American Society of Clinical Oncologists/National Comprehensive Cancer Network and the European Society of Breast Cancer Specialists.^18,19^ We selected measures applicable to treatments available at BCCOE and adapted these based on the nature of available data (Table 1).

**Table 1:**
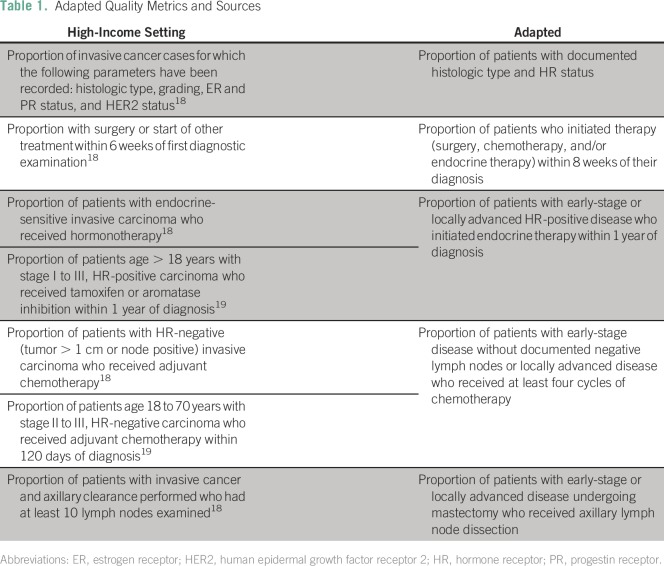
Adapted Quality Metrics and Sources

Additionally, we developed a measure to assess receipt of three recommended treatments for those with early-stage or locally advanced disease: surgery, at least four cycles of chemotherapy, and initiation of endocrine therapy within 1 year of first biopsy. Patients whose planned chemotherapy was truncated after disease progression (ie, progression, recurrence, or death within 180 days of their last treatment) were excluded from consideration. Thus, patients were defined as having not completed treatment if they were lost from care for at least 180 days (including progression, recurrence, or death at > 180 days from last documented treatment) or entirely before completing indicated treatment.

### Chemotherapy Dose-Intensity

We calculated the delivered and relative dose-intensities of chemotherapy as a function of dose and the time over which it was administered. Delivered dose-intensity only considers administered chemotherapy and measures the extent to which toxicities and logistical challenges delayed or limited chemotherapy administration.^24^ Relative dose-intensity includes consideration of planned cycles that were missed entirely, thereby capturing dose-intensity reductions resulting from incomplete treatment or loss to follow-up. Receipt of a relative dose-intensity of at least 0.85 corresponds with greater likelihood of breast cancer survival.^25^

All dose-intensity calculations relied on the summation dose-intensity method outlined by Hryniuk et al.^26^ If a patient only received neoadjuvant or adjuvant chemotherapy, all indicated chemotherapy was evaluated as if planned for neoadjuvant or adjuvant delivery, respectively. If a patient received both neoadjuvant and adjuvant treatments, the number of neoadjuvant cycles received was considered the planned number, and the remainder of specified cycles was considered planned for adjuvant delivery. In cases of progression or death during curative-intent chemotherapy, planned cycles only included those administered before progression. When planned cycles were missed, a dose of 0 mg/m^2^ and the standard cycle length were recorded.

### Analysis

In our cohort, patient age, stage at presentation, tumor histologic type and grade, estrogen and progesterone receptor (HR) status, HER2 status, and treatments received were analyzed using descriptive statistics. The five patients without a determined stage were excluded from analyses stratified by stage. For each quality measure, we identified the percentage of eligible patients whose care was concordant with the measure.

To accommodate missing data regarding timing of death, as described in Key Variables, we used an interval-censored survival analysis strategy to determine overall survival rates. An expectation-maximization iterative convex minorant algorithm was used to determine the nonparametric maximum likelihood estimator of the survival functions of the cohort.^27,28^ All analyses were performed using SAS software (version 9.4; SAS Institute, Cary, NC).

### Ethics

Ethical approval for this study was obtained from the Partners HealthCare Institutional Review Board (Boston, MA) and the Rwanda National Ethics Committee.

## RESULTS

### Patient and Tumor Characteristics

A total of 162 patients received a pathologic diagnosis of breast cancer during the study period. Four male patients, four patients diagnosed with ductal carcinoma in situ only, and three patients whose medical records could not be located were excluded, leaving 150 patients in the final analysis. The median age was 48 years. Twenty-eight patients were diagnosed with early-stage disease, 64 with locally advanced disease, and 53 with metastatic disease, and five had no documented disease stage (Table 2). The most common histologic type was invasive ductal carcinoma. Of 148 patients with known HR status, 67.6% had HR-positive disease. HER2 status was not routinely assessed for all patients, because directed therapies were not available as a result of cost. However, of 38 patients with known HER2 status, 26% had HER2-positive tumors (Table 2).

**Table 2:**
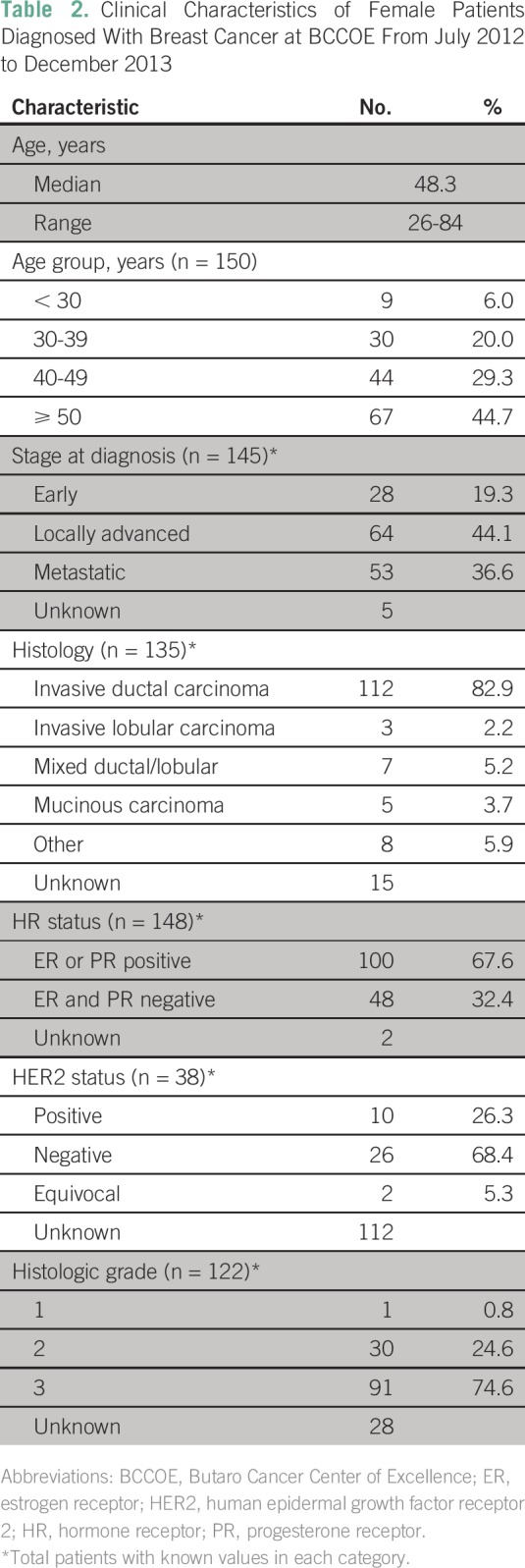
Clinical Characteristics of Female Patients Diagnosed With Breast Cancer at BCCOE From July 2012 to December 2013

### Treatments and Care Quality

Twenty-one patients with early-stage disease (75.0%) and 37 with locally advanced disease (57.8%) underwent breast surgery (Table 3). The mean time to surgery from biopsy or completion of neoadjuvant chemotherapy was 68 days (range, 0 to 434 days). Only 35.4% of potentially curable patients (ie, those with early-stage or locally advanced disease) received surgery within 60 days of first biopsy or end of neoadjuvant chemotherapy.

**Table 3:**
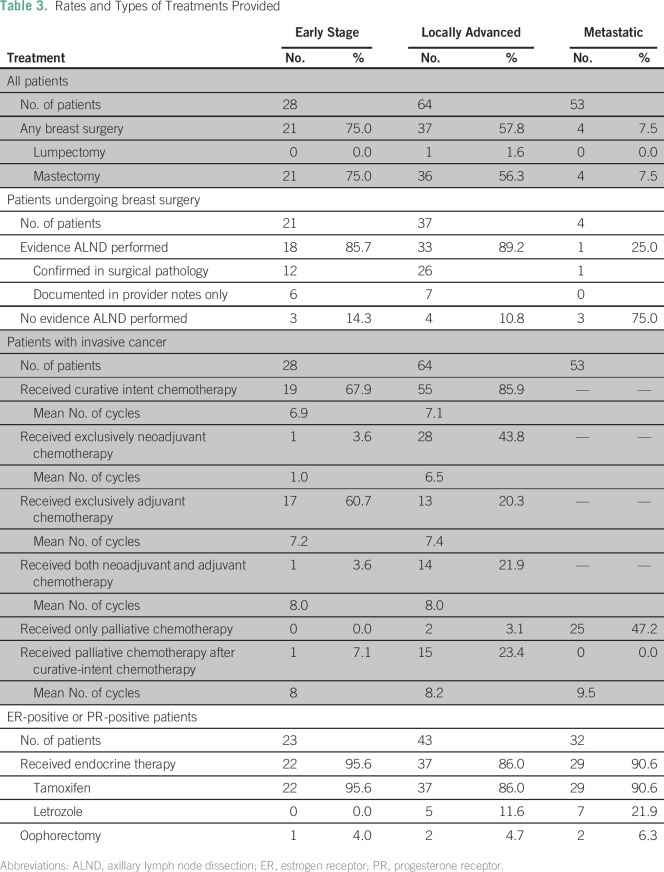
Rates and Types of Treatments Provided

Among patients with early-stage disease, all but one had an indication for chemotherapy based on national protocols, and 67.9% received chemotherapy. Of eight cycles most commonly recommended, the mean number delivered was 6.9 (range, one to eight). In the locally advanced group, 85.9% received chemotherapy, with a mean of 7.1 cycles (range, one to 12 cycles; Table 3). Twenty-five patients (47.2%) with metastatic cancers received palliative chemotherapy (Table 3). Among HR-positive patients, 95.6% of those with early-stage disease and 86.0% of those with locally advanced disease initiated endocrine therapy (Table 3).

Among patients with early-stage or locally advanced cancers, mean delivered dose-intensities, which consider only administered chemotherapy, were 0.93 and 0.95 for neoadjuvant and adjuvant chemotherapy, respectively. Relative dose-intensity, which considers administered and planned chemotherapy, was greater than 0.85 in 50% of patients with early-stage disease and 61.9% of those with locally advanced disease receiving neoadjuvant chemotherapy and 83.3% of those with early-stage disease and 81.5% of those with locally advanced disease receiving adjuvant chemotherapy (Table 4).

**Table 4:**
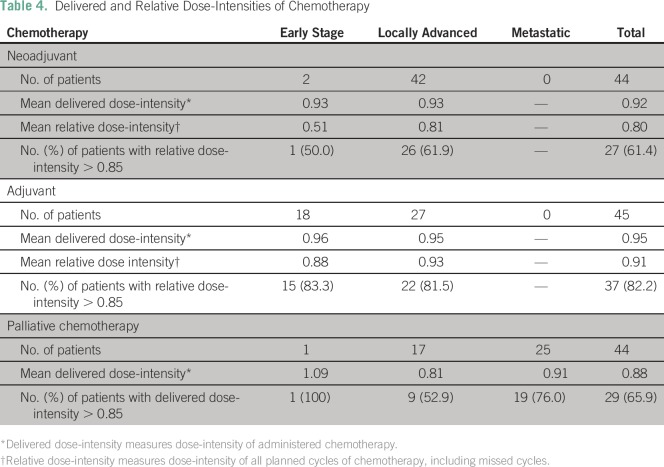
Delivered and Relative Dose-Intensities of Chemotherapy

The most common causes for chemotherapy delay were neutropenia, patients missing appointments, infection, and provider or hospital delays. The most common reasons for chemotherapy dose reductions were neuropathy and neutropenia (Data Supplement). The most commonly documented chemotherapy toxicities were neutropenia, nausea, neuropathy, and vomiting (Data Supplement).

Among all 150 patients, rates of documented histologic tumor type and HR status were high (98.0%). Three quarters (74.7%) of patients initiated treatment within 8 weeks of biopsy. Most patients (83.3% of all HR-positive patients) received endocrine therapy within 1 year of biopsy. Nearly three quarters of eligible potentially curable patients (73.6%) received four or more cycles of curative-intent chemotherapy. Of patients undergoing breast surgery, 87.9% had evidence of ALND (Table 5).

**Table 5:**
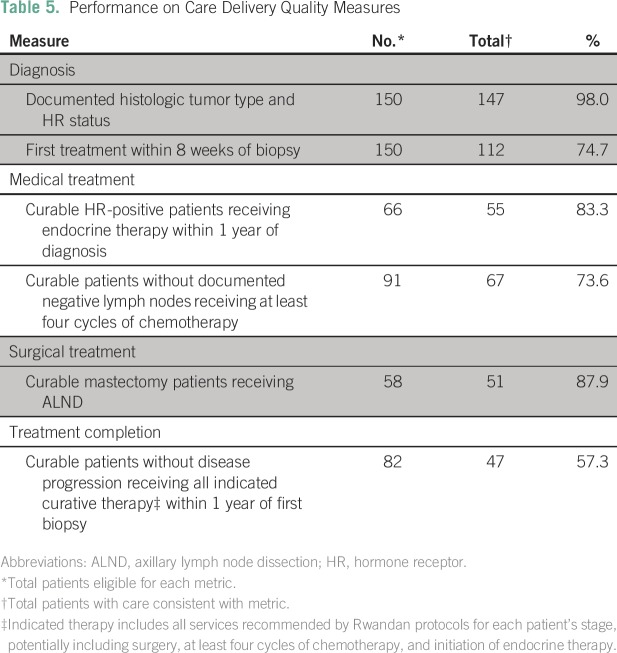
Performance on Care Delivery Quality Measures

Among potentially curable patients who had no evidence of progression, 57.3% received all indicated therapy (ie, surgery, chemotherapy, and/or initiation of endocrine therapy) within 1 year of diagnosis (Table 5). The most frequently missing modality was surgery, with 31 patients having no documented surgery within 1 year of diagnosis. Reasons for the lack of documented surgery were often not clear.

### Outcomes

**Fig 1:**
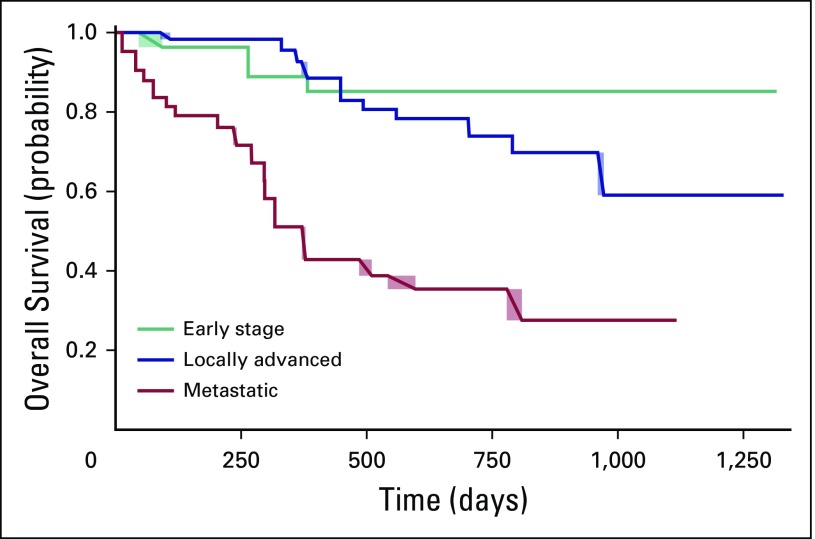
Overall survival by disease stage at diagnosis.

Median follow-up time was 18.3 months for all patients and 24.2 months for potentially curable patients. Among the 28 patients with early-stage disease, 23 (82.1%) were alive at the end of follow-up, four (14.3%) had died, and one (3.6%) had been lost to follow-up. Among the 64 patients with locally advanced disease, 34 (53.1%) were alive, 18 (28.1%) had died, and 12 (18.8%) had been lost to follow-up. Finally, among the 53 patients with metastatic disease, eight (15.1%) were alive, 29 (54.7%) had died, and 16 (30.2%) had been lost to follow-up. The median survival for the metastatic group fell between 10.6 and 12.4 months. Figure 1 displays the interval-censored survival curves for each stage.

## DISCUSSION

We examined the quality of breast cancer care provided in a rural public cancer facility in Rwanda that operates through partnership between the government, an international nongovernmental organization, and academic institutions. In this setting, the quality of chemotherapy and endocrine therapy was high, whereas rates of timely and appropriate surgical care were suboptimal.

Rates of chemotherapy receipt were high; 74% of eligible patients received at least four cycles of chemotherapy. This compares favorably to other African centers, where reported adherence rates range from 29% to 84%.^8,11,29^ The mean delivered dose-intensity of curative-intent chemotherapy was greater than 0.90. This is notable, because the incidence of grade 3 or 4 neutropenia was comparable to rates in other published studies despite the lack of granulocyte colony-stimulating factor support.^30-32^ When we included planned chemotherapy, the proportion of patients receiving a relative dose-intensity greater than 0.85 was comparable to rates in recent reports in North America and superior to rates from the 1990s.^34,35^ Receipt of endocrine therapy was also high. More than 85% of all patients with HR-positive tumors initiated endocrine therapy, with nearly all starting within 1 year of diagnosis.

Critically, provision of surgery was far less consistent in this early cohort. Only 63% of potentially curable patients underwent breast surgery. Reported mastectomy rates in other sub-Saharan African centers range from 75% to 95% of patients.^11,29,36^ The relatively low rates of surgery that we observed are partially explained by the infrequent availability of onsite surgery in the early months after the opening of BCCOE, requiring referral to Rwanda’s overburdened teaching hospitals. Retrospective studies in the United States have suggested an association between undergoing surgery within 60 days of diagnosis and longer survival,^37^ but only 35% of potentially curable patients at BCCOE received surgery within this timeframe. In mid-2013, a full-time general surgeon was hired at BCCOE and received specialized training from Boston-based oncologic surgeons. Patient volume, staff turnover, and insurance reimbursement policies have meant that patients sometimes still require referral to other hospitals for surgery. Because inadequate follow-up ensuring that surgery is performed has hampered many referrals, stronger patient navigation systems are being developed. Future analyses should examine the impact of onsite surgical services and enhanced support of patients referred elsewhere on receipt of timely breast surgery.

Low surgery rates and loss to care were the main reasons for suboptimal rates of treatment completion, with only 57% of potentially curable patients receiving all indicated curative therapies. Community health workers in Rwanda have been highly effective in retaining patients with HIV in care.^38^ They have not been leveraged yet for cancer care, but they may be a future resource. Fuller understanding of the logistical, financial, and cultural barriers to care faced by patients at BCCOE will be critical to developing targeted interventions to improve treatment completion rates.

To be meaningful and pragmatic, quality process metrics should rely on routinely collected data, address processes with potential for improvement, and they should be based on evidence showing correlation with outcomes.^20,39^ Our adapted metrics met the first two criteria. All data were taken from clinical documents, and no process, aside from pathology documentation, approached universal performance. Studying the association between quality metric performance and clinical outcomes in these settings is an important next step. Given the low surgical rates in our study, simple receipt of surgery may be an essential additional metric to track in the low-resource context.

Care quality can also be judged by comparing patient outcomes with those at similar centers. The median survival at BCCOE for patients with early-stage or locally advanced disease exceeded 3.6 years. These results compare favorably to those at other centers in Africa. Reports from Uganda show survival probabilities of 100% among patients with stage I or II disease and 52% for those with stage III disease at 36 months.^40^ An Ethiopian cohort had rates of metastasis-free survival at 2 years of 85% in patients with stage I or II disease and 66% in those with stage III disease.^29^ Unsurprisingly, BCCOE survival rates were inferior to those in the United States, where the 5-year survival probability is 99% for stage I cancers and 85% for stage II to IIIb cancers.^41^ Although lack of radiotherapy and HER2-targeting agents likely contribute to this difference, our results suggest that improvements in breast cancer survival could be achieved through improving the quality of and retention in currently available care, particularly regarding access to surgery.

Our findings have several limitations. First, although data quality was excellent for medical therapies, surgical data were suboptimal. It is possible that some patients who were lost to follow-up on referral for surgery actually underwent mastectomy and that surgical rates were higher than reported. Second, computed tomography was used much less frequently for staging than in high-resource settings, potentially resulting in a greater proportion of patients having undetected metastatic disease at presentation and limiting comparison with outcomes in high-resource settings. Third, our survival analysis assumed that all censoring was noninformative. However, patients with late-stage disease were more likely to be lost to follow-up, and the cohort of censored patients was likely at a higher risk for death. Nonetheless, our overall loss to follow-up rate of 20% compares favorably to rates in other breast cancer studies from sub-Saharan Africa, allowing comparison with regional literature.^11,29,40^ Finally, we focused on a single facility receiving heavy investment from governmental and international partners. Ongoing work will determine whether our results are generalizable to other centers.

This study demonstrates that delivery of complete, appropriate dose-intensity chemotherapy and prompt initiation of endocrine therapy are possible in a low-resource setting in sub-Saharan Africa. Prompt access to surgery was a major issue for the women diagnosed during the first 18 months after the opening of BCCOE. Overall early survival rates compare favorably to other limited evidence from the region. Future studies are planned to examine factors facilitating high-quality care and assess the association between higher care quality and survival. These findings can guide continued investment in breast cancer care in Rwanda and other sub-Saharan African nations.
